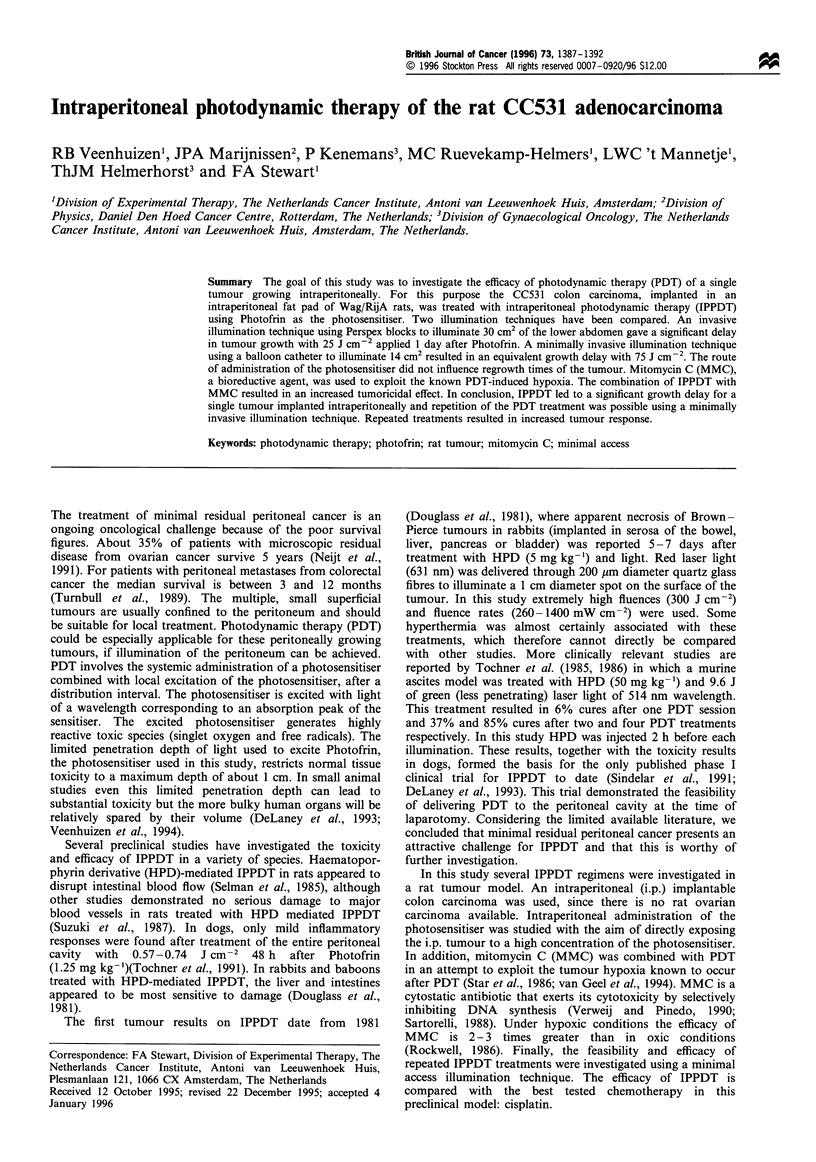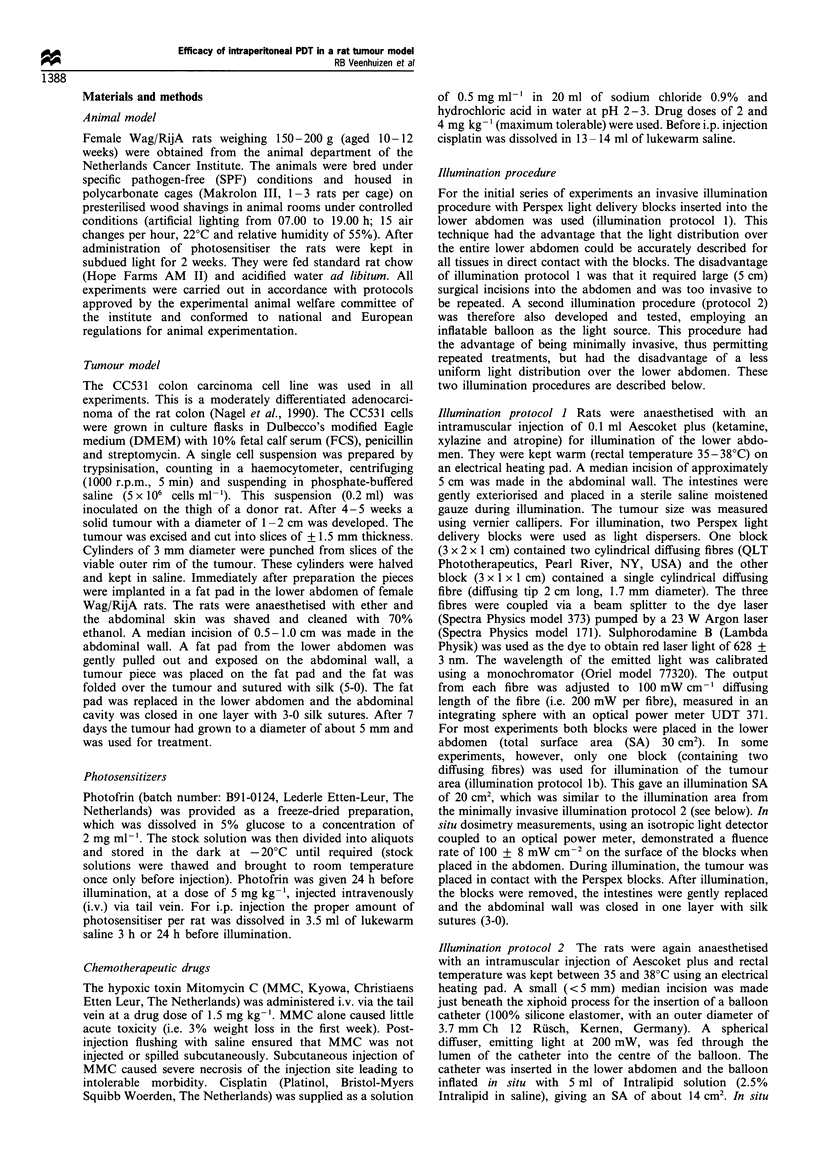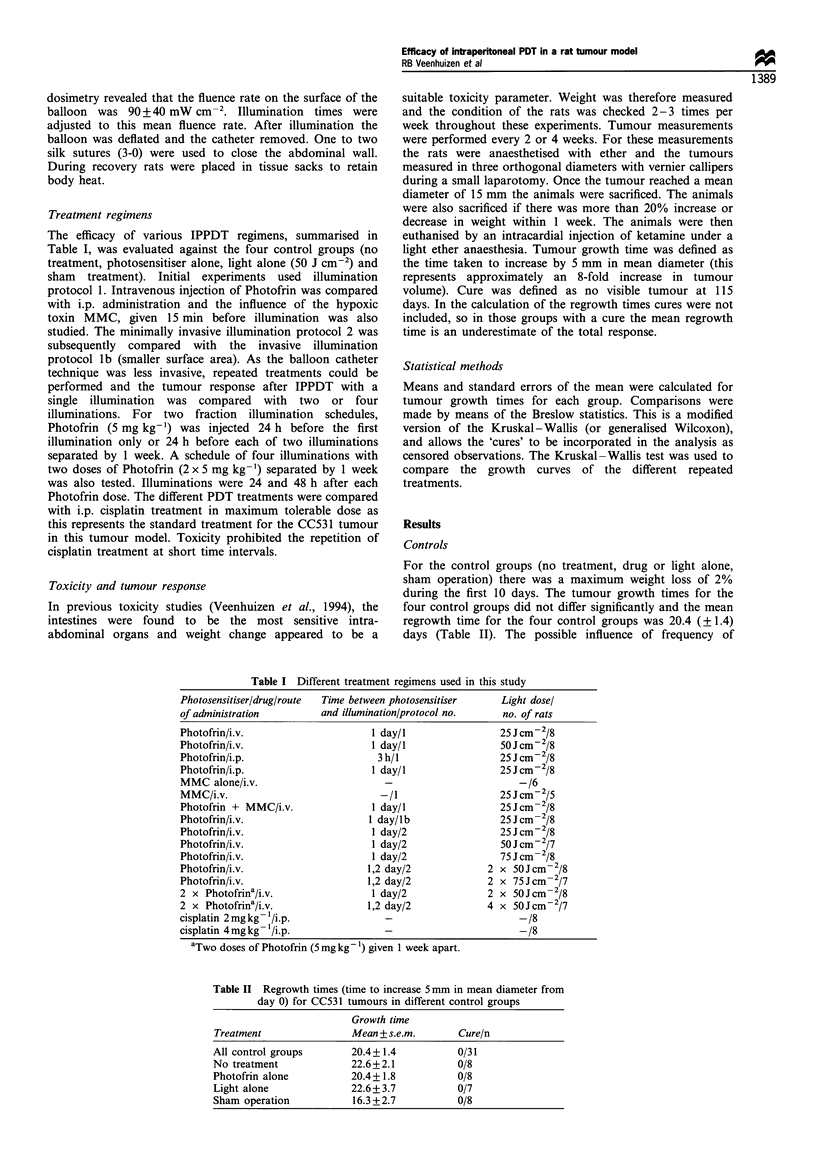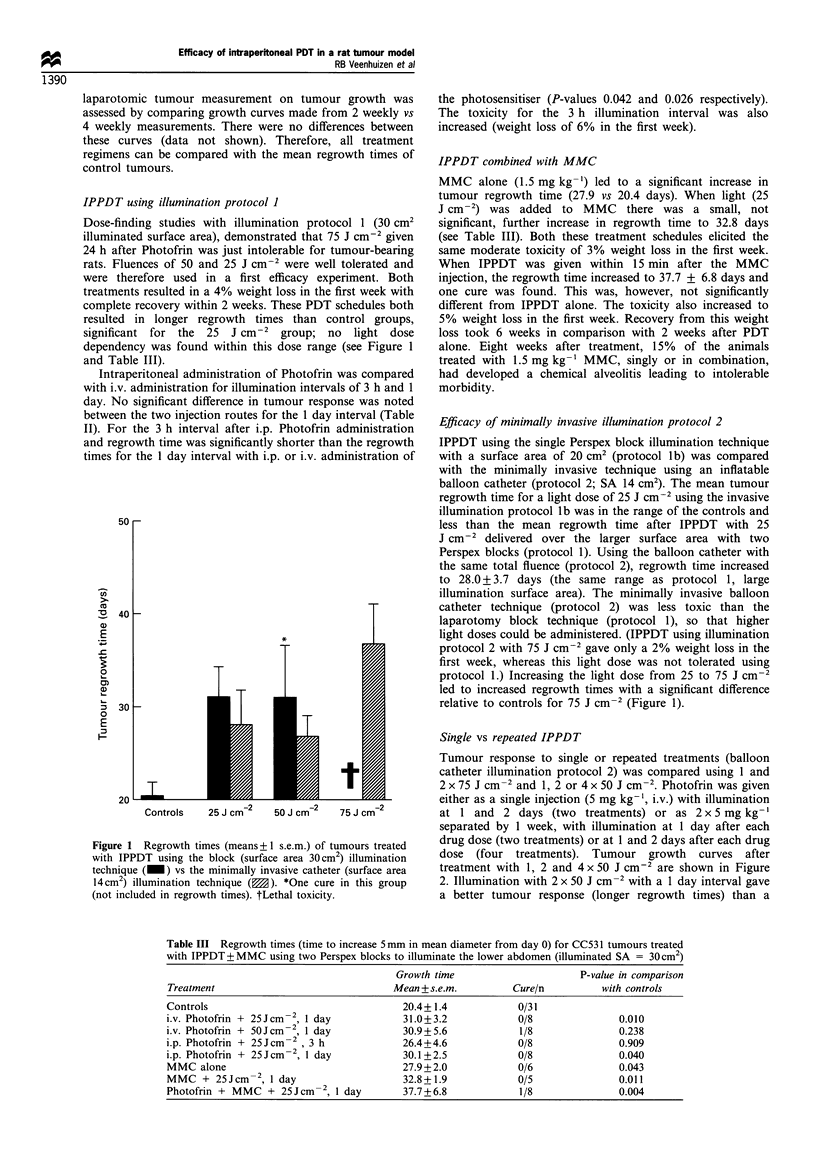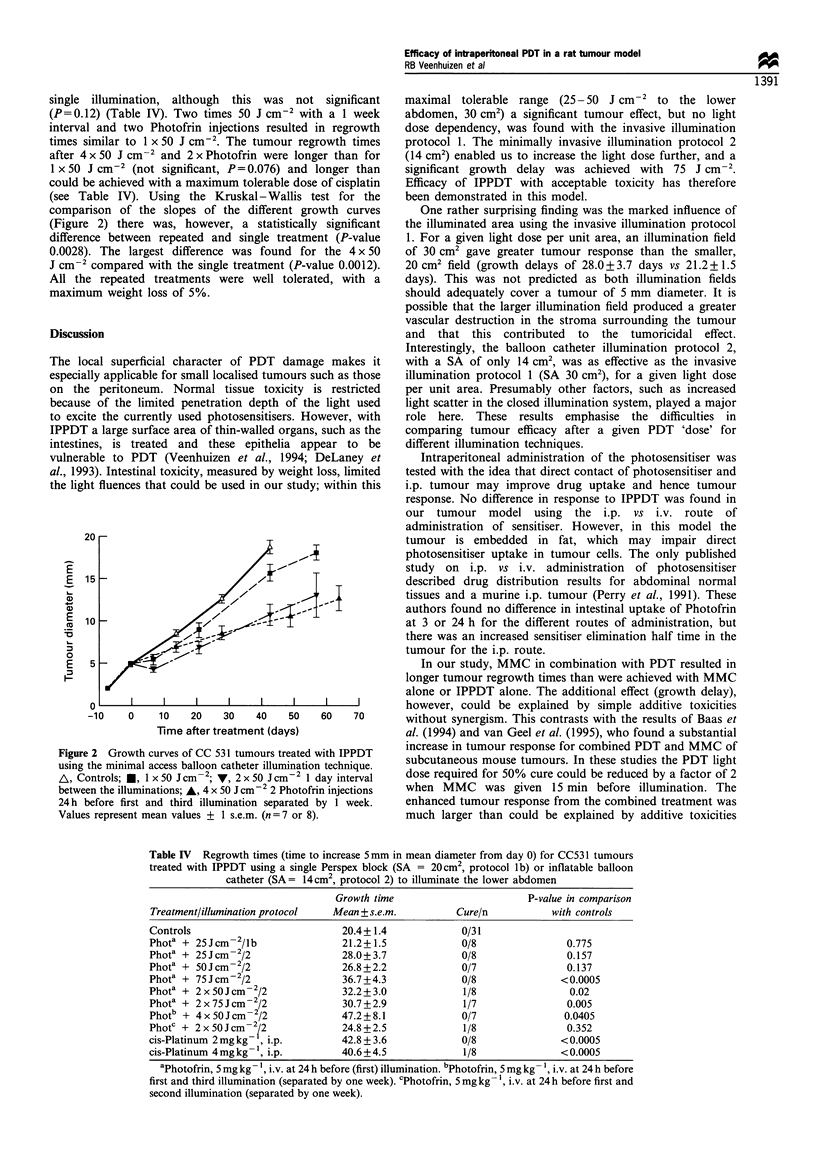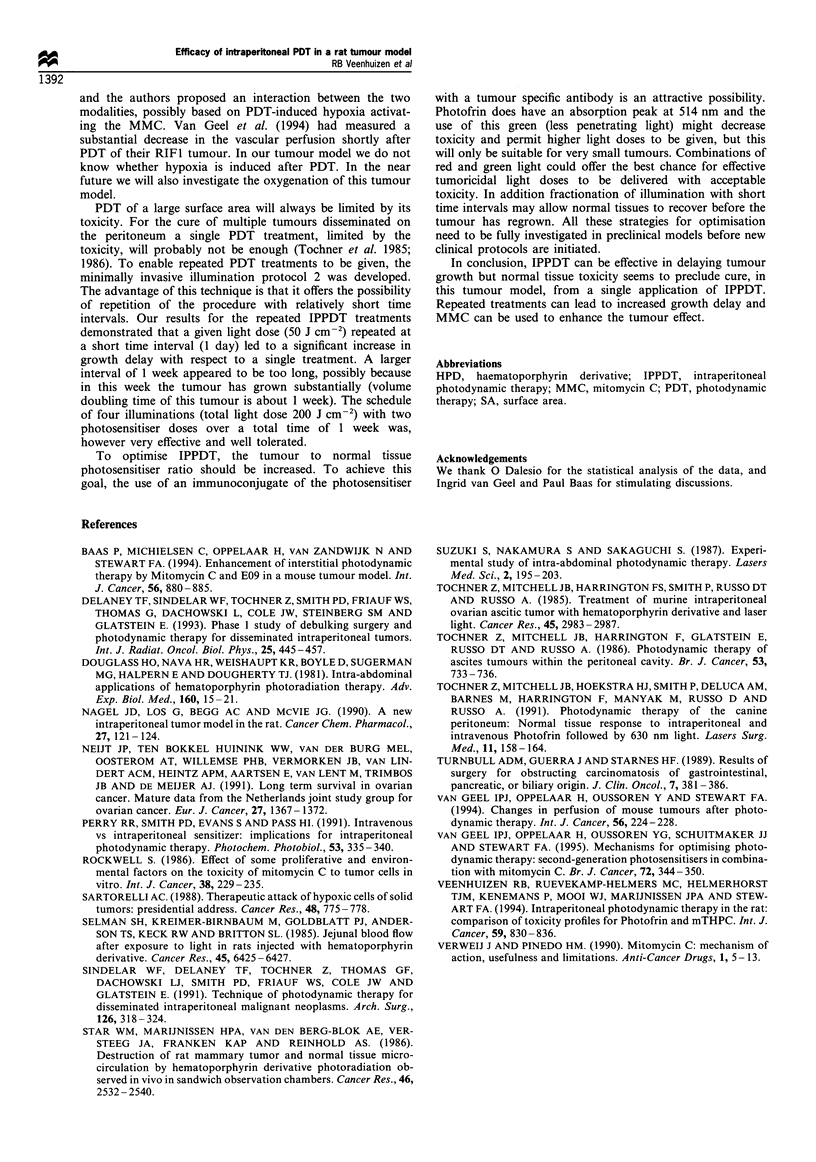# Intraperitoneal photodynamic therapy of the rat CC531 adenocarcinoma.

**DOI:** 10.1038/bjc.1996.263

**Published:** 1996-06

**Authors:** R. B. Veenhuizen, J. P. Marijnissen, P. Kenemans, M. C. Ruevekamp-Helmers, L. W. 't Mannetje, T. J. Helmerhorst, F. A. Stewart

**Affiliations:** Division of Experimental Therapy, The Netherlands Cancer Institute, Amsterdam, The Netherlands.

## Abstract

The goal of this study was to investigate the efficacy of photodynamic therapy (PDT) of a single tumour growing intraperitoneally. For this purpose the CC531 colon carcinoma, implanted in an intraperitoneal fat pad of Wag/RijA rats, was treated with intraperitoneal photodynamic therapy (IPPDT) using Photofrin as the photosensitiser. Two illumination techniques have been compared. An invasive illumination technique using Perspex blocks to illuminate 30 cm2 of the lower abdomen gave a significant delay in tumour growth with 25 J cm-2 applied 1 day after Photofrin. A minimally invasive illumination technique using a balloon catheter to illuminate 14 cm2 resulted in an equivalent growth delay with 75 J cm-2. The route of administration of the photosensitiser did not influence regrowth times of the tumour. Mitomycin C (MMC), a bioreductive agent, was used to exploit the known PDT-induced hypoxia. The combination of IPPDT with MMC resulted in an increased tumoricidal effect. In conclusion, IPPDT led to a significant growth delay for a single tumour implanted intraperitoneally and repetition of the PDT treatment was possible using a minimally invasive illumination technique. Repeated treatments resulted in increased tumour response.


British Journal of Cancer (1996) 73, 1387-1392
© 1996 Stockton Press All rights reserved 0007-0920/96 $12.00

### Intraperitoneal photodynamic therapy of the rat CC531 adenocarcinoma

RB Veenhuizen[1], JPA Marijnissen[2], P Kenemans[3], MC Ruevekamp-Helmers[1], LWC 't Mannetje[1], ThJM Helmerhorst[3] and FA Stewart[1]

[1]*Division of Experimental Therapy, The Netherlands Cancer Institute, Antoni van Leeuwenhoek Huis, Amsterdam;* [2]*Division of Physics, Daniel Den Hoed Cancer Centre, Rotterdam, The Netherlands;* [3]*Division of Gynaecological Oncology, The Netherlands Cancer Institute, Antoni van Leeuwenhoek Huis, Amsterdam, The Netherlands.*

**Summary** The goal of this study was to investigate the efficacy of photodynamic therapy (PDT) of a single tumour growing intraperitoneally. For this purpose the CC531 colon carcinoma, implanted in an intraperitoneal fat pad of Wag/RijA rats, was treated with intraperitoneal photodynamic therapy (IPPDT) using Photofrin as the photosensitiser. Two illumination techniques have been compared. An invasive illumination technique using Perspex blocks to illuminate 30 $cm^2$ of the lower abdomen gave a significant delay in tumour growth with 25 J $cm^{-2}$ applied 1 day after Photofrin. A minimally invasive illumination technique using a balloon catheter to illuminate 14 $cm^2$ resulted in an equivalent growth delay with 75 J $cm^{-2}$. The route of administration of the photosensitiser did not influence regrowth times of the tumour. Mitomycin C (MMC), a bioreductive agent, was used to exploit the known PDT-induced hypoxia. The combination of IPPDT with MMC resulted in an increased tumoricidal effect. In conclusion, IPPDT led to a significant growth delay for a single tumour implanted intraperitoneally and repetition of the PDT treatment was possible using a minimally invasive illumination technique. Repeated treatments resulted in increased tumour response.

**Keywords:** photodynamic therapy; photofrin; rat tumour; mitomycin C; minimal access

The treatment of minimal residual peritoneal cancer is an ongoing oncological challenge because of the poor survival figures. About 35% of patients with microscopic residual disease from ovarian cancer survive 5 years (Neijt *et al.*, 1991). For patients with peritoneal metastases from colorectal cancer the median survival is between 3 and 12 months (Turnbull *et al.*, 1989). The multiple, small superficial tumours are usually confined to the peritoneum and should be suitable for local treatment. Photodynamic therapy (PDT) could be especially applicable for these peritoneally growing tumours, if illumination of the peritoneum can be achieved. PDT involves the systemic administration of a photosensitiser combined with local excitation of the photosensitiser, after a distribution interval. The photosensitiser is excited with light of a wavelength corresponding to an absorption peak of the sensitiser. The excited photosensitiser generates highly reactive toxic species (singlet oxygen and free radicals). The limited penetration depth of light used to excite Photofrin, the photosensitiser used in this study, restricts normal tissue toxicity to a maximum depth of about 1 cm. In small animal studies even this limited penetration depth can lead to substantial toxicity but the more bulky human organs will be relatively spared by their volume (DeLaney *et al.*, 1993; Veenhuizen *et al.*, 1994).

Several preclinical studies have investigated the toxicity and efficacy of IPPDT in a variety of species. Haematoporphyrin derivative (HPD)-mediated IPPDT in rats appeared to disrupt intestinal blood flow (Selman *et al.*, 1985), although other studies demonstrated no serious damage to major blood vessels in rats treated with HPD mediated IPPDT (Suzuki *et al.*, 1987). In dogs, only mild inflammatory responses were found after treatment of the entire peritoneal cavity with 0.57–0.74 J $cm^{-2}$ 48 h after Photofrin (1.25 mg $kg^{-1}$)(Tochner *et al.*, 1991). In rabbits and baboons treated with HPD-mediated IPPDT, the liver and intestines appeared to be most sensitive to damage (Douglass *et al.*, 1981).

The first tumour results on IPPDT date from 1981 (Douglass *et al.*, 1981), where apparent necrosis of Brown–Pierce tumours in rabbits (implanted in serosa of the bowel, liver, pancreas or bladder) was reported 5–7 days after treatment with HPD (5 mg $kg^{-1}$) and light. Red laser light (631 nm) was delivered through 200 $\mu$m diameter quartz glass fibres to illuminate a 1 cm diameter spot on the surface of the tumour. In this study extremely high fluences (300 J $cm^{-2}$) and fluence rates (260–1400 mW $cm^{-2}$) were used. Some hyperthermia was almost certainly associated with these treatments, which therefore cannot directly be compared with other studies. More clinically relevant studies are reported by Tochner *et al.* (1985, 1986) in which a murine ascites model was treated with HPD (50 mg $kg^{-1}$) and 9.6 J of green (less penetrating) laser light of 514 nm wavelength. This treatment resulted in 6% cures after one PDT session and 37% and 85% cures after two and four PDT treatments respectively. In this study HPD was injected 2 h before each illumination. These results, together with the toxicity results in dogs, formed the basis for the only published phase I clinical trial for IPPDT to date (Sindelar *et al.*, 1991; DeLaney *et al.*, 1993). This trial demonstrated the feasibility of delivering PDT to the peritoneal cavity at the time of laparotomy. Considering the limited available literature, we concluded that minimal residual peritoneal cancer presents an attractive challenge for IPPDT and that this is worthy of further investigation.

In this study several IPPDT regimens were investigated in a rat tumour model. An intraperitoneal (i.p.) implantable colon carcinoma was used, since there is no rat ovarian carcinoma available. Intraperitoneal administration of the photosensitiser was studied with the aim of directly exposing the i.p. tumour to a high concentration of the photosensitiser. In addition, mitomycin C (MMC) was combined with PDT in an attempt to exploit the tumour hypoxia known to occur after PDT (Star *et al.*, 1986; van Geel *et al.*, 1994). MMC is a cytostatic antibiotic that exerts its cytotoxicity by selectively inhibiting DNA synthesis (Verweij and Pinedo, 1990; Sartorelli, 1988). Under hypoxic conditions the efficacy of MMC is 2–3 times greater than in oxic conditions (Rockwell, 1986). Finally, the feasibility and efficacy of repeated IPPDT treatments were investigated using a minimal access illumination technique. The efficacy of IPPDT is compared with the best tested chemotherapy in this preclinical model: cisplatin.

Correspondence: FA Stewart, Division of Experimental Therapy, The Netherlands Cancer Institute, Antoni van Leeuwenhoek Huis, Plesmanlaan 121, 1066 CX Amsterdam, The Netherlands
Received 12 October 1995; revised 22 December 1995; accepted 4 January 1996

### Materials and methods

### Animal model

Female Wag/RijA rats weighing 150–200 g (aged 10–12 weeks) were obtained from the animal department of the Netherlands Cancer Institute. The animals were bred under specific pathogen-free (SPF) conditions and housed in polycarbonate cages (Makrolon III, 1–3 rats per cage) on presterilised wood shavings in animal rooms under controlled conditions (artificial lighting from 07.00 to 19.00 h; 15 air changes per hour, 22°C and relative humidity of 55%). After administration of photosensitiser the rats were kept in subdued light for 2 weeks. They were fed standard rat chow (Hope Farms AM II) and acidified water *ad libitum*. All experiments were carried out in accordance with protocols approved by the experimental animal welfare committee of the institute and conformed to national and European regulations for animal experimentation.

### Tumour model

The CC531 colon carcinoma cell line was used in all experiments. This is a moderately differentiated adenocarcinoma of the rat colon (Nagel *et al.*, 1990). The CC531 cells were grown in culture flasks in Dulbecco's modified Eagle medium (DMEM) with 10% fetal calf serum (FCS), penicillin and streptomycin. A single cell suspension was prepared by trypsinisation, counting in a haemocytometer, centrifuging (1000 r.p.m., 5 min) and suspending in phosphate-buffered saline ($5 \times 10^6$ cells $ml^{-1}$). This suspension (0.2 ml) was inoculated on the thigh of a donor rat. After 4–5 weeks a solid tumour with a diameter of 1–2 cm was developed. The tumour was excised and cut into slices of $\pm 1.5$ mm thickness. Cylinders of 3 mm diameter were punched from slices of the viable outer rim of the tumour. These cylinders were halved and kept in saline. Immediately after preparation the pieces were implanted in a fat pad in the lower abdomen of female Wag/RijA rats. The rats were anaesthetised with ether and the abdominal skin was shaved and cleaned with 70% ethanol. A median incision of 0.5–1.0 cm was made in the abdominal wall. A fat pad from the lower abdomen was gently pulled out and exposed on the abdominal wall, a tumour piece was placed on the fat pad and the fat was folded over the tumour and sutured with silk (5-0). The fat pad was replaced in the lower abdomen and the abdominal cavity was closed in one layer with 3-0 silk sutures. After 7 days the tumour had grown to a diameter of about 5 mm and was used for treatment.

### Photosensitizers

Photofrin (batch number: B91-0124, Lederle Etten-Leur, The Netherlands) was provided as a freeze-dried preparation, which was dissolved in 5% glucose to a concentration of 2 mg $ml^{-1}$. The stock solution was then divided into aliquots and stored in the dark at $-20$°C until required (stock solutions were thawed and brought to room temperature once only before injection). Photofrin was given 24 h before illumination, at a dose of 5 mg $kg^{-1}$, injected intravenously (i.v.) via tail vein. For i.p. injection the proper amount of photosensitiser per rat was dissolved in 3.5 ml of lukewarm saline 3 h or 24 h before illumination.

### Chemotherapeutic drugs

The hypoxic toxin Mitomycin C (MMC, Kyowa, Christiaens Etten Leur, The Netherlands) was administered i.v. via the tail vein at a drug dose of 1.5 mg $kg^{-1}$. MMC alone caused little acute toxicity (i.e. 3% weight loss in the first week). Post-injection flushing with saline ensured that MMC was not injected or spilled subcutaneously. Subcutaneous injection of MMC caused severe necrosis of the injection site leading to intolerable morbidity. Cisplatin (Platinol, Bristol-Myers Squibb Woerden, The Netherlands) was supplied as a solution of 0.5 mg $ml^{-1}$ in 20 ml of sodium chloride 0.9% and hydrochloric acid in water at pH 2–3. Drug doses of 2 and 4 mg $kg^{-1}$ (maximum tolerable) were used. Before i.p. injection cisplatin was dissolved in 13–14 ml of lukewarm saline.

### Illumination procedure

For the initial series of experiments an invasive illumination procedure with Perspex light delivery blocks inserted into the lower abdomen was used (illumination protocol 1). This technique had the advantage that the light distribution over the entire lower abdomen could be accurately described for all tissues in direct contact with the blocks. The disadvantage of illumination protocol 1 was that it required large (5 cm) surgical incisions into the abdomen and was too invasive to be repeated. A second illumination procedure (protocol 2) was therefore also developed and tested, employing an inflatable balloon as the light source. This procedure had the advantage of being minimally invasive, thus permitting repeated treatments, but had the disadvantage of a less uniform light distribution over the lower abdomen. These two illumination procedures are described below.

*Illumination protocol 1* Rats were anaesthetised with an intramuscular injection of 0.1 ml Aescoket plus (ketamine, xylazine and atropine) for illumination of the lower abdomen. They were kept warm (rectal temperature 35–38°C) on an electrical heating pad. A median incision of approximately 5 cm was made in the abdominal wall. The intestines were gently exteriorised and placed in a sterile saline moistened gauze during illumination. The tumour size was measured using vernier callipers. For illumination, two Perspex light delivery blocks were used as light dispersers. One block ($3 \times 2 \times 1$ cm) contained two cylindrical diffusing fibres (QLT Phototherapeutics, Pearl River, NY, USA) and the other block ($3 \times 1 \times 1$ cm) contained a single cylindrical diffusing fibre (diffusing tip 2 cm long, 1.7 mm diameter). The three fibres were coupled via a beam splitter to the dye laser (Spectra Physics model 373) pumped by a 23 W Argon laser (Spectra Physics model 171). Sulphorodamine B (Lambda Physik) was used as the dye to obtain red laser light of 628 $\pm$ 3 nm. The wavelength of the emitted light was calibrated using a monochromator (Oriel model 77320). The output from each fibre was adjusted to 100 mW $cm^{-1}$ diffusing length of the fibre (i.e. 200 mW per fibre), measured in an integrating sphere with an optical power meter UDT 371. For most experiments both blocks were placed in the lower abdomen (total surface area (SA) 30 $cm^2$). In some experiments, however, only one block (containing two diffusing fibres) was used for illumination of the tumour area (illumination protocol 1b). This gave an illumination SA of 20 $cm^2$, which was similar to the illumination area from the minimally invasive illumination protocol 2 (see below). *In situ* dosimetry measurements, using an isotropic light detector coupled to an optical power meter, demonstrated a fluence rate of 100 $\pm$ 8 mW $cm^{-2}$ on the surface of the blocks when placed in the abdomen. During illumination, the tumour was placed in contact with the Perspex blocks. After illumination, the blocks were removed, the intestines were gently replaced and the abdominal wall was closed in one layer with silk sutures (3-0).

*Illumination protocol 2* The rats were again anaesthetised with an intramuscular injection of Aescoket plus and rectal temperature was kept between 35 and 38°C using an electrical heating pad. A small (< 5 mm) median incision was made just beneath the xiphoid process for the insertion of a balloon catheter (100% silicone elastomer, with an outer diameter of 3.7 mm Ch 12 Rüsch, Kernen, Germany). A spherical diffuser, emitting light at 200 mW, was fed through the lumen of the catheter into the centre of the balloon. The catheter was inserted in the lower abdomen and the balloon inflated *in situ* with 5 ml of Intralipid solution (2.5% Intralipid in saline), giving an SA of about 14 $cm^2$. *In situ*

dosimetry revealed that the fluence rate on the surface of the balloon was $90 \pm 40$ mW $cm^{-2}$. Illumination times were adjusted to this mean fluence rate. After illumination the balloon was deflated and the catheter removed. One to two silk sutures (3-0) were used to close the abdominal wall. During recovery rats were placed in tissue sacks to retain body heat.

*Treatment regimens*

The efficacy of various IPPDT regimens, summarised in Table I, was evaluated against the four control groups (no treatment, photosensitiser alone, light alone (50 J $cm^{-2}$) and sham treatment). Initial experiments used illumination protocol 1. Intravenous injection of Photofrin was compared with i.p. administration and the influence of the hypoxic toxin MMC, given 15 min before illumination was also studied. The minimally invasive illumination protocol 2 was subsequently compared with the invasive illumination protocol 1b (smaller surface area). As the balloon catheter technique was less invasive, repeated treatments could be performed and the tumour response after IPPDT with a single illumination was compared with two or four illuminations. For two fraction illumination schedules, Photofrin (5 mg $kg^{-1}$) was injected 24 h before the first illumination only or 24 h before each of two illuminations separated by 1 week. A schedule of four illuminations with two doses of Photofrin ($2 \times 5$ mg $kg^{-1}$) separated by 1 week was also tested. Illuminations were 24 and 48 h after each Photofrin dose. The different PDT treatments were compared with i.p. cisplatin treatment in maximum tolerable dose as this represents the standard treatment for the CC531 tumour in this tumour model. Toxicity prohibited the repetition of cisplatin treatment at short time intervals.

*Toxicity and tumour response*

In previous toxicity studies (Veenhuizen *et al.*, 1994), the intestines were found to be the most sensitive intra-abdominal organs and weight change appeared to be a suitable toxicity parameter. Weight was therefore measured and the condition of the rats was checked 2–3 times per week throughout these experiments. Tumour measurements were performed every 2 or 4 weeks. For these measurements the rats were anaesthetised with ether and the tumours measured in three orthogonal diameters with vernier callipers during a small laparotomy. Once the tumour reached a mean diameter of 15 mm the animals were sacrificed. The animals were also sacrificed if there was more than 20% increase or decrease in weight within 1 week. The animals were then euthanised by an intracardial injection of ketamine under a light ether anaesthesia. Tumour growth time was defined as the time taken to increase by 5 mm in mean diameter (this represents approximately an 8-fold increase in tumour volume). Cure was defined as no visible tumour at 115 days. In the calculation of the regrowth times cures were not included, so in those groups with a cure the mean regrowth time is an underestimate of the total response.

*Statistical methods*

Means and standard errors of the mean were calculated for tumour growth times for each group. Comparisons were made by means of the Breslow statistics. This is a modified version of the Kruskal–Wallis (or generalised Wilcoxon), and allows the 'cures' to be incorporated in the analysis as censored observations. The Kruskal–Wallis test was used to compare the growth curves of the different repeated treatments.

### Results

*Controls*

For the control groups (no treatment, drug or light alone, sham operation) there was a maximum weight loss of 2% during the first 10 days. The tumour growth times for the four control groups did not differ significantly and the mean regrowth time for the four control groups was 20.4 ($\pm 1.4$) days (Table II). The possible influence of frequency of

**Table I** Different treatment regimens used in this study

| *Photosensitiser/drug/route of administration* | *Time between photosensitiser and illumination/protocol no.* | *Light dose/ no. of rats* |
|---|---|---|
| Photofrin/i.v. | 1 day/1 | 25 J $cm^{-2}$/8 |
| Photofrin/i.v. | 1 day/1 | 50 J $cm^{-2}$/8 |
| Photofrin/i.p. | 3 h/1 | 25 J $cm^{-2}$/8 |
| Photofrin/i.p. | 1 day/1 | 25 J $cm^{-2}$/8 |
| MMC alone/i.v. | – | –/6 |
| MMC/i.v. | –/1 | 25 J $cm^{-2}$/5 |
| Photofrin + MMC/i.v. | 1 day/1 | 25 J $cm^{-2}$/8 |
| Photofrin/i.v. | 1 day/1b | 25 J $cm^{-2}$/8 |
| Photofrin/i.v. | 1 day/2 | 25 J $cm^{-2}$/8 |
| Photofrin/i.v. | 1 day/2 | 50 J $cm^{-2}$/7 |
| Photofrin/i.v. | 1 day/2 | 75 J $cm^{-2}$/8 |
| Photofrin/i.v. | 1,2 day/2 | 2 × 50 J $cm^{-2}$/8 |
| Photofrin/i.v. | 1,2 day/2 | 2 × 75 J $cm^{-2}$/7 |
| 2 × Photofrin[a]/i.v. | 1 day/2 | 2 × 50 J $cm^{-2}$/8 |
| 2 × Photofrin[a]/i.v. | 1,2 day/2 | 4 × 50 J $cm^{-2}$/7 |
| cisplatin 2 mg $kg^{-1}$/i.p. | – | –/8 |
| cisplatin 4 mg $kg^{-1}$/i.p. | – | –/8 |

[a]Two doses of Photofrin (5 mg $kg^{-1}$) given 1 week apart.

**Table II** Regrowth times (time to increase 5 mm in mean diameter from day 0) for CC531 tumours in different control groups

| *Treatment* | *Growth time Mean ± s.e.m.* | *Cure/n* |
|---|---|---|
| All control groups | 20.4 ± 1.4 | 0/31 |
| No treatment | 22.6 ± 2.1 | 0/8 |
| Photofrin alone | 20.4 ± 1.8 | 0/8 |
| Light alone | 22.6 ± 3.7 | 0/7 |
| Sham operation | 16.3 ± 2.7 | 0/8 |

laparotomic tumour measurement on tumour growth was assessed by comparing growth curves made from 2 weekly *vs* 4 weekly measurements. There were no differences between these curves (data not shown). Therefore, all treatment regimens can be compared with the mean regrowth times of control tumours.

### *IPPDT using illumination protocol 1*

Dose-finding studies with illumination protocol 1 (30 $cm^2$ illuminated surface area), demonstrated that 75 J $cm^{-2}$ given 24 h after Photofrin was just intolerable for tumour-bearing rats. Fluences of 50 and 25 J $cm^{-2}$ were well tolerated and were therefore used in a first efficacy experiment. Both treatments resulted in a 4% weight loss in the first week with complete recovery within 2 weeks. These PDT schedules both resulted in longer regrowth times than control groups, significant for the 25 J $cm^{-2}$ group; no light dose dependency was found within this dose range (see Figure 1 and Table III).

Intraperitoneal administration of Photofrin was compared with i.v. administration for illumination intervals of 3 h and 1 day. No significant difference in tumour response was noted between the two injection routes for the 1 day interval (Table II). For the 3 h interval after i.p. Photofrin administration and regrowth time was significantly shorter than the regrowth times for the 1 day interval with i.p. or i.v. administration of the photosensitiser (*P*-values 0.042 and 0.026 respectively). The toxicity for the 3 h illumination interval was also increased (weight loss of 6% in the first week).

**Figure 1** Regrowth times (means ± 1 s.e.m.) of tumours treated with IPPDT using the block (surface area 30 $cm^2$) illumination technique (■) vs the minimally invasive catheter (surface area 14 $cm^2$) illumination technique (▨). *One cure in this group (not included in regrowth times). †Lethal toxicity.

### *IPPDT combined with MMC*

MMC alone (1.5 mg $kg^{-1}$) led to a significant increase in tumour regrowth time (27.9 *vs* 20.4 days). When light (25 J $cm^{-2}$) was added to MMC there was a small, not significant, further increase in regrowth time to 32.8 days (see Table III). Both these treatment schedules elicited the same moderate toxicity of 3% weight loss in the first week. When IPPDT was given within 15 min after the MMC injection, the regrowth time increased to 37.7 ± 6.8 days and one cure was found. This was, however, not significantly different from IPPDT alone. The toxicity also increased to 5% weight loss in the first week. Recovery from this weight loss took 6 weeks in comparison with 2 weeks after PDT alone. Eight weeks after treatment, 15% of the animals treated with 1.5 mg $kg^{-1}$ MMC, singly or in combination, had developed a chemical alveolitis leading to intolerable morbidity.

### *Efficacy of minimally invasive illumination protocol 2*

IPPDT using the single Perspex block illumination technique with a surface area of 20 $cm^2$ (protocol 1b) was compared with the minimally invasive technique using an inflatable balloon catheter (protocol 2; SA 14 $cm^2$). The mean tumour regrowth time for a light dose of 25 J $cm^{-2}$ using the invasive illumination protocol 1b was in the range of the controls and less than the mean regrowth time after IPPDT with 25 J $cm^{-2}$ delivered over the larger surface area with two Perspex blocks (protocol 1). Using the balloon catheter with the same total fluence (protocol 2), regrowth time increased to 28.0 ± 3.7 days (the same range as protocol 1, large illumination surface area). The minimally invasive balloon catheter technique (protocol 2) was less toxic than the laparotomy block technique (protocol 1), so that higher light doses could be administered. (IPPDT using illumination protocol 2 with 75 J $cm^{-2}$ gave only a 2% weight loss in the first week, whereas this light dose was not tolerated using protocol 1.) Increasing the light dose from 25 to 75 J $cm^{-2}$ led to increased regrowth times with a significant difference relative to controls for 75 J $cm^{-2}$ (Figure 1).

### *Single* vs *repeated IPPDT*

Tumour response to single or repeated treatments (balloon catheter illumination protocol 2) was compared using 1 and 2 × 75 J $cm^{-2}$ and 1, 2 or 4 × 50 J $cm^{-2}$. Photofrin was given either as a single injection (5 mg $kg^{-1}$, i.v.) with illumination at 1 and 2 days (two treatments) or as 2 × 5 mg $kg^{-1}$ separated by 1 week, with illumination at 1 day after each drug dose (two treatments) or at 1 and 2 days after each drug dose (four treatments). Tumour growth curves after treatment with 1, 2 and 4 × 50 J $cm^{-2}$ are shown in Figure 2. Illumination with 2 × 50 J $cm^{-2}$ with a 1 day interval gave a better tumour response (longer regrowth times) than a

**Table III** Regrowth times (time to increase 5 mm in mean diameter from day 0) for CC531 tumours treated with IPPDT ± MMC using two Perspex blocks to illuminate the lower abdomen (illuminated SA = 30 $cm^2$)

| *Treatment* | *Growth time Mean ± s.e.m.* | *Cure/n* | *P-value in comparison with controls* |
|---|---|---|---|
| Controls | 20.4 ± 1.4 | 0/31 | |
| i.v. Photofrin + 25 J $cm^{-2}$, 1 day | 31.0 ± 3.2 | 0/8 | 0.010 |
| i.v. Photofrin + 50 J $cm^{-2}$, 1 day | 30.9 ± 5.6 | 1/8 | 0.238 |
| i.p. Photofrin + 25 J $cm^{-2}$, 3 h | 26.4 ± 4.6 | 0/8 | 0.909 |
| i.p. Photofrin + 25 J $cm^{-2}$, 1 day | 30.1 ± 2.5 | 0/8 | 0.040 |
| MMC alone | 27.9 ± 2.0 | 0/6 | 0.043 |
| MMC + 25 J $cm^{-2}$, 1 day | 32.8 ± 1.9 | 0/5 | 0.011 |
| Photofrin + MMC + 25 J $cm^{-2}$, 1 day | 37.7 ± 6.8 | 1/8 | 0.004 |

single illumination, although this was not significant ($P=0.12$) (Table IV). Two times 50 J cm$^{-2}$ with a 1 week interval and two Photofrin injections resulted in regrowth times similar to $1\times 50$ J cm$^{-2}$. The tumour regrowth times after $4\times 50$ J cm$^{-2}$ and $2\times$Photofrin were longer than for $1\times 50$ J cm$^{-2}$ (not significant, $P=0.076$) and longer than could be achieved with a maximum tolerable dose of cisplatin (see Table IV). Using the Kruskal–Wallis test for the comparison of the slopes of the different growth curves (Figure 2) there was, however, a statistically significant difference between repeated and single treatment ($P$-value 0.0028). The largest difference was found for the $4\times 50$ J cm$^{-2}$ compared with the single treatment ($P$-value 0.0012). All the repeated treatments were well tolerated, with a maximum weight loss of 5%.

### Discussion

The local superficial character of PDT damage makes it especially applicable for small localised tumours such as those on the peritoneum. Normal tissue toxicity is restricted because of the limited penetration depth of the light used to excite the currently used photosensitisers. However, with IPPDT a large surface area of thin-walled organs, such as the intestines, is treated and these epithelia appear to be vulnerable to PDT (Veenhuizen *et al.*, 1994; DeLaney *et al.*, 1993). Intestinal toxicity, measured by weight loss, limited the light fluences that could be used in our study; within this maximal tolerable range (25–50 J cm$^{-2}$ to the lower abdomen, 30 cm$^2$) a significant tumour effect, but no light dose dependency, was found with the invasive illumination protocol 1. The minimally invasive illumination protocol 2 (14 cm$^2$) enabled us to increase the light dose further, and a significant growth delay was achieved with 75 J cm$^{-2}$. Efficacy of IPPDT with acceptable toxicity has therefore been demonstrated in this model.

**Figure 2** Growth curves of CC 531 tumours treated with IPPDT using the minimal access balloon catheter illumination technique. △, Controls; ■, $1\times 50$ J cm$^{-2}$; ▼, $2\times 50$ J cm$^{-2}$ 1 day interval between the illuminations; ▲, $4\times 50$ J cm$^{-2}$ 2 Photofrin injections 24 h before first and third illumination separated by 1 week. Values represent mean values ± 1 s.e.m. ($n=7$ or 8).

One rather surprising finding was the marked influence of the illuminated area using the invasive illumination protocol 1. For a given light dose per unit area, an illumination field of 30 cm$^2$ gave greater tumour response than the smaller, 20 cm$^2$ field (growth delays of 28.0±3.7 days *vs* 21.2±1.5 days). This was not predicted as both illumination fields should adequately cover a tumour of 5 mm diameter. It is possible that the larger illumination field produced a greater vascular destruction in the stroma surrounding the tumour and that this contributed to the tumoricidal effect. Interestingly, the balloon catheter illumination protocol 2, with a SA of only 14 cm$^2$, was as effective as the invasive illumination protocol 1 (SA 30 cm$^2$), for a given light dose per unit area. Presumably other factors, such as increased light scatter in the closed illumination system, played a major role here. These results emphasise the difficulties in comparing tumour efficacy after a given PDT 'dose' for different illumination techniques.

Intraperitoneal administration of the photosensitiser was tested with the idea that direct contact of photosensitiser and i.p. tumour may improve drug uptake and hence tumour response. No difference in response to IPPDT was found in our tumour model using the i.p. *vs* i.v. route of administration of sensitiser. However, in this model the tumour is embedded in fat, which may impair direct photosensitiser uptake in tumour cells. The only published study on i.p. *vs* i.v. administration of photosensitiser described drug distribution results for abdominal normal tissues and a murine i.p. tumour (Perry *et al.*, 1991). These authors found no difference in intestinal uptake of Photofrin at 3 or 24 h for the different routes of administration, but there was an increased sensitiser elimination half time in the tumour for the i.p. route.

In our study, MMC in combination with PDT resulted in longer tumour regrowth times than were achieved with MMC alone or IPPDT alone. The additional effect (growth delay), however, could be explained by simple additive toxicities without synergism. This contrasts with the results of Baas *et al.* (1994) and van Geel *et al.* (1995), who found a substantial increase in tumour response for combined PDT and MMC of subcutaneous mouse tumours. In these studies the PDT light dose required for 50% cure could be reduced by a factor of 2 when MMC was given 15 min before illumination. The enhanced tumour response from the combined treatment was much larger than could be explained by additive toxicities

**Table IV** Regrowth times (time to increase 5 mm in mean diameter from day 0) for CC531 tumours treated with IPPDT using a single Perspex block (SA = 20 cm$^2$, protocol 1b) or inflatable balloon catheter (SA = 14 cm$^2$, protocol 2) to illuminate the lower abdomen

| *Treatment/illumination protocol* | *Growth time Mean±s.e.m.* | *Cure/n* | *P-value in comparison with controls* |
|---|---|---|---|
| Controls | 20.4±1.4 | 0/31 | |
| Phot[a] + 25 J cm$^{-2}$/1b | 21.2±1.5 | 0/8 | 0.775 |
| Phot[a] + 25 J cm$^{-2}$/2 | 28.0±3.7 | 0/8 | 0.157 |
| Phot[a] + 50 J cm$^{-2}$/2 | 26.8±2.2 | 0/7 | 0.137 |
| Phot[a] + 75 J cm$^{-2}$/2 | 36.7±4.3 | 0/8 | <0.0005 |
| Phot[a] + 2×50 J cm$^{-2}$/2 | 32.2±3.0 | 1/8 | 0.02 |
| Phot[a] + 2×75 J cm$^{-2}$/2 | 30.7±2.9 | 1/7 | 0.005 |
| Phot[b] + 4×50 J cm$^{-2}$/2 | 47.2±8.1 | 0/7 | 0.0405 |
| Phot[c] + 2×50 J cm$^{-2}$/2 | 24.8±2.5 | 1/8 | 0.352 |
| cis-Platinum 2 mg kg$^{-1}$, i.p. | 42.8±3.6 | 0/8 | <0.0005 |
| cis-Platinum 4 mg kg$^{-1}$, i.p. | 40.6±4.5 | 1/8 | <0.0005 |

[a]Photofrin, 5 mg kg$^{-1}$, i.v. at 24 h before (first) illumination. [b]Photofrin, 5 mg kg$^{-1}$, i.v. at 24 h before first and third illumination (separated by one week). [c]Photofrin, 5 mg kg$^{-1}$, i.v. at 24 h before first and second illumination (separated by one week).

and the authors proposed an interaction between the two modalities, possibly based on PDT-induced hypoxia activating the MMC. Van Geel *et al.* (1994) had measured a substantial decrease in the vascular perfusion shortly after PDT of their RIF1 tumour. In our tumour model we do not know whether hypoxia is induced after PDT. In the near future we will also investigate the oxygenation of this tumour model.

PDT of a large surface area will always be limited by its toxicity. For the cure of multiple tumours disseminated on the peritoneum a single PDT treatment, limited by the toxicity, will probably not be enough (Tochner *et al.* 1985; 1986). To enable repeated PDT treatments to be given, the minimally invasive illumination protocol 2 was developed. The advantage of this technique is that it offers the possibility of repetition of the procedure with relatively short time intervals. Our results for the repeated IPPDT treatments demonstrated that a given light dose (50 J $cm^{-2}$) repeated at a short time interval (1 day) led to a significant increase in growth delay with respect to a single treatment. A larger interval of 1 week appeared to be too long, possibly because in this week the tumour has grown substantially (volume doubling time of this tumour is about 1 week). The schedule of four illuminations (total light dose 200 J $cm^{-2}$) with two photosensitiser doses over a total time of 1 week was, however very effective and well tolerated.

To optimise IPPDT, the tumour to normal tissue photosensitiser ratio should be increased. To achieve this goal, the use of an immunoconjugate of the photosensitiser with a tumour specific antibody is an attractive possibility. Photofrin does have an absorption peak at 514 nm and the use of this green (less penetrating light) might decrease toxicity and permit higher light doses to be given, but this will only be suitable for very small tumours. Combinations of red and green light could offer the best chance for effective tumoricidal light doses to be delivered with acceptable toxicity. In addition fractionation of illumination with short time intervals may allow normal tissues to recover before the tumour has regrown. All these strategies for optimisation need to be fully investigated in preclinical models before new clinical protocols are initiated.

In conclusion, IPPDT can be effective in delaying tumour growth but normal tissue toxicity seems to preclude cure, in this tumour model, from a single application of IPPDT. Repeated treatments can lead to increased growth delay and MMC can be used to enhance the tumour effect.

### Abbreviations

HPD, haematoporphyrin derivative; IPPDT, intraperitoneal photodynamic therapy; MMC, mitomycin C; PDT, photodynamic therapy; SA, surface area.

### Acknowledgements

We thank O Dalesio for the statistical analysis of the data, and Ingrid van Geel and Paul Baas for stimulating discussions.

### References

BAAS P, MICHIELSEN C, OPPELAAR H, VAN ZANDWIJK N AND STEWART FA. (1994). Enhancement of interstitial photodynamic therapy by Mitomycin C and E09 in a mouse tumour model. *Int. J. Cancer*, **56**, 880–885.

DELANEY TF, SINDELAR WF, TOCHNER Z, SMITH PD, FRIAUF WS, THOMAS G, DACHOWSKI L, COLE JW, STEINBERG SM AND GLATSTEIN E. (1993). Phase 1 study of debulking surgery and photodynamic therapy for disseminated intraperitoneal tumors. *Int. J. Radiat. Oncol. Biol. Phys.*, **25**, 445–457.

DOUGLASS HO, NAVA HR, WEISHAUPT KR, BOYLE D, SUGERMAN MG, HALPERN E AND DOUGHERTY TJ. (1981). Intra-abdominal applications of hematoporphyrin photoradiation therapy. *Adv. Exp. Biol. Med.*, **160**, 15–21.

NAGEL JD, LOS G, BEGG AC AND McVIE JG. (1990). A new intraperitoneal tumor model in the rat. *Cancer Chem. Pharmacol.*, **27**, 121–124.

NEIJT JP, TEN BOKKEL HUININK WW, VAN DER BURG MEL, OOSTEROM AT, WILLEMSE PHB, VERMORKEN JB, VAN LINDERT ACM, HEINTZ APM, AARTSEN E, VAN LENT M, TRIMBOS JB AND DE MEIJER AJ. (1991). Long term survival in ovarian cancer. Mature data from the Netherlands joint study group for ovarian cancer. *Eur. J. Cancer*, **27**, 1367–1372.

PERRY RR, SMITH PD, EVANS S AND PASS HI. (1991). Intravenous vs intraperitoneal sensitizer: implications for intraperitoneal photodynamic therapy. *Photochem. Photobiol.*, **53**, 335–340.

ROCKWELL S. (1986). Effect of some proliferative and environmental factors on the toxicity of mitomycin C to tumor cells in vitro. *Int. J. Cancer*, **38**, 229–235.

SARTORELLI AC. (1988). Therapeutic attack of hypoxic cells of solid tumors: presidential address. *Cancer Res.*, **48**, 775–778.

SELMAN SH, KREIMER-BIRNBAUM M, GOLDBLATT PJ, ANDERSON TS, KECK RW AND BRITTON SL. (1985). Jejunal blood flow after exposure to light in rats injected with hematoporphyrin derivative. *Cancer Res.*, **45**, 6425–6427.

SINDELAR WF, DELANEY TF, TOCHNER Z, THOMAS GF, DACHOWSKI LJ, SMITH PD, FRIAUF WS, COLE JW AND GLATSTEIN E. (1991). Technique of photodynamic therapy for disseminated intraperitoneal malignant neoplasms. *Arch. Surg.*, **126**, 318–324.

STAR WM, MARIJNISSEN HPA, VAN DEN BERG-BLOK AE, VERSTEEG JA, FRANKEN KAP AND REINHOLD AS. (1986). Destruction of rat mammary tumor and normal tissue microcirculation by hematoporphyrin derivative photoradiation observed in vivo in sandwich observation chambers. *Cancer Res.*, **46**, 2532–2540.

SUZUKI S, NAKAMURA S AND SAKAGUCHI S. (1987). Experimental study of intra-abdominal photodynamic therapy. *Lasers Med. Sci.*, **2**, 195–203.

TOCHNER Z, MITCHELL JB, HARRINGTON FS, SMITH P, RUSSO DT AND RUSSO A. (1985). Treatment of murine intraperitoneal ovarian ascitic tumor with hematoporphyrin derivative and laser light. *Cancer Res.*, **45**, 2983–2987.

TOCHNER Z, MITCHELL JB, HARRINGTON F, GLATSTEIN E, RUSSO DT AND RUSSO A. (1986). Photodynamic therapy of ascites tumours within the peritoneal cavity. *Br. J. Cancer*, **53**, 733–736.

TOCHNER Z, MITCHELL JB, HOEKSTRA HJ, SMITH P, DELUCA AM, BARNES M, HARRINGTON F, MANYAK M, RUSSO D AND RUSSO A. (1991). Photodynamic therapy of the canine peritoneum: Normal tissue response to intraperitoneal and intravenous Photofrin followed by 630 nm light. *Lasers Surg. Med.*, **11**, 158–164.

TURNBULL ADM, GUERRA J AND STARNES HF. (1989). Results of surgery for obstructing carcinomatosis of gastrointestinal, pancreatic, or biliary origin. *J. Clin. Oncol.*, **7**, 381–386.

VAN GEEL IPJ, OPPELAAR H, OUSSOREN Y AND STEWART FA. (1994). Changes in perfusion of mouse tumours after photodynamic therapy. *Int. J. Cancer*, **56**, 224–228.

VAN GEEL IPJ, OPPELAAR H, OUSSOREN YG, SCHUITMAKER JJ AND STEWART FA. (1995). Mechanisms for optimising photodynamic therapy: second-generation photosensitisers in combination with mitomycin C. *Br. J. Cancer*, **72**, 344–350.

VEENHUIZEN RB, RUEVEKAMP-HELMERS MC, HELMERHORST TJM, KENEMANS P, MOOI WJ, MARIJNISSEN JPA AND STEWART FA. (1994). Intraperitoneal photodynamic therapy in the rat: comparison of toxicity profiles for Photofrin and mTHPC. *Int. J. Cancer*, **59**, 830–836.

VERWEIJ J AND PINEDO HM. (1990). Mitomycin C: mechanism of action, usefulness and limitations. *Anti-Cancer Drugs*, **1**, 5–13.